# The Clinician's Guide to the Machine Learning Galaxy

**DOI:** 10.3389/fphys.2021.658583

**Published:** 2021-04-06

**Authors:** Lin Shen, Benjamin H. Kann, R. Andrew Taylor, Dennis L. Shung

**Affiliations:** ^1^Department of Medicine, Brigham and Women's Hospital, Boston, MA, United States; ^2^Division of Gastroenterology, Hepatology and Endoscopy, Brigham and Women's Hospital, Boston, MA, United States; ^3^Department of Radiation Oncology, Dana-Farber Cancer Institute/Brigham and Women's Hospital and Harvard Medical School, Boston, MA, United States; ^4^Artificial Intelligence in Medicine Program, Brigham and Women's Hospital, Boston, MA, United States; ^5^Department of Emergency Medicine, Yale School of Medicine, New Haven, CT, United States; ^6^Section of Digestive Diseases, Department of Medicine, Yale School of Medicine, New Haven, CT, United States

**Keywords:** artificial intelligence, delivery of health care, machine learning, clinical decision support systems, health care outcome and process assessment

## Introduction

Machine learning has the potential to enhance the practice of medicine (Rajkomar et al., 2019). However, an “AI chasm” has been described that limit the clinical application of machine learning models (Keane and Topol, 2018). Clinicians are domain experts that can help bridge the gap by becoming active partners in developing and implementing machine learning models for clinical use. The paradigm of collaboration between domain experts and machine learning engineers has been successful in developing expert-augmented machine learning (Gennatas et al., 2020). However, it is challenging for interested clinicians to understand the capabilities of machine learning and how to best contribute their domain expertise in designing a machine learning solution.

**Figure 1:**
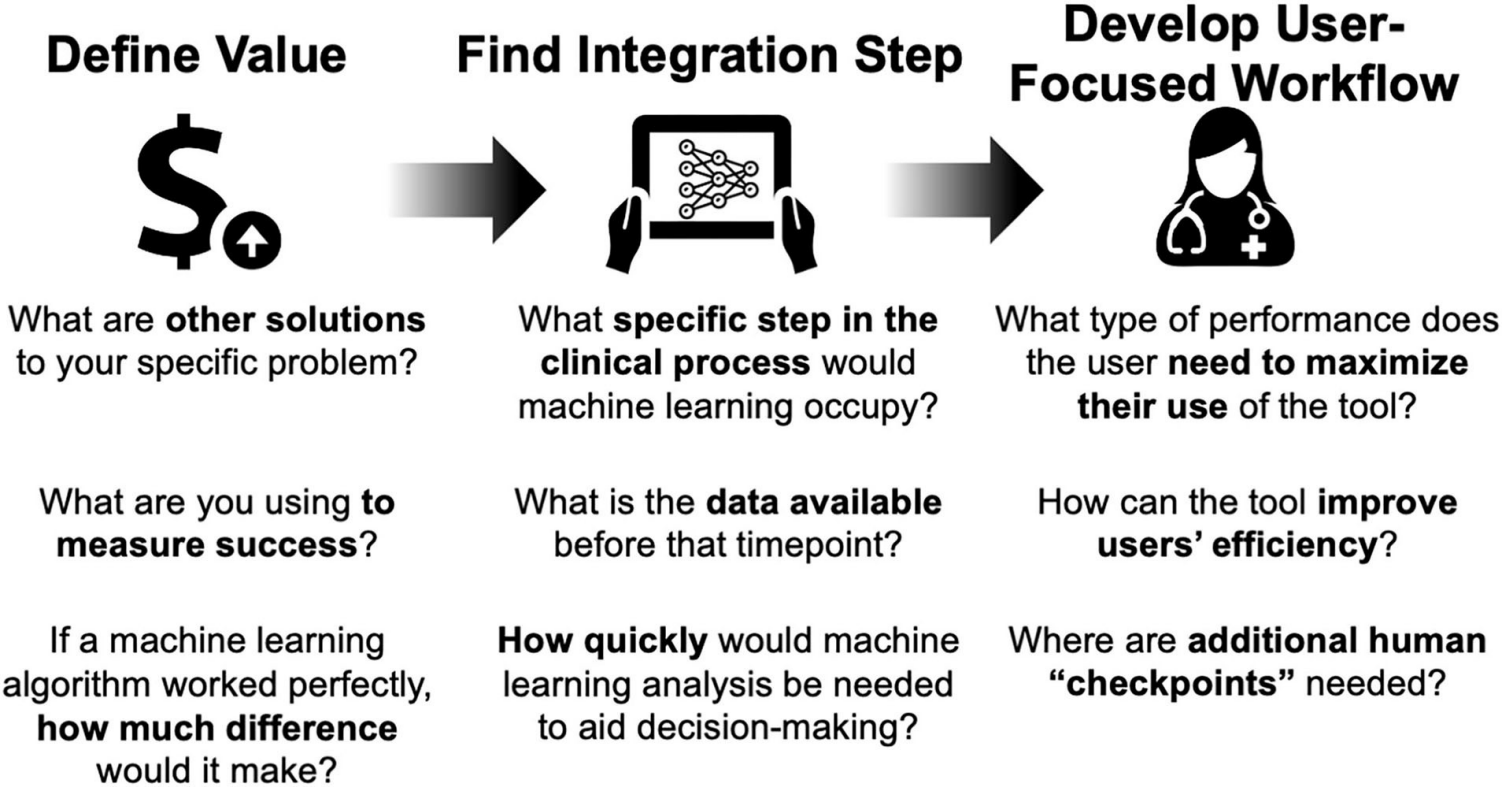
Framework for considering artificial intelligence-based tools for clinical care.

This is a guide for the clinician interested in helping to design and deploy machine learning solutions to improve clinical care. We propose an approach that finds an area with potential for benefit, considers machine learning as one of several solutions, then counts the cost of a perfectly performing machine learning algorithm to determine if it is worth the effort (Figure 1).

### Key Terms

***Artificial intelligence (AI):*** Generally, the ability for a computer to accomplish tasks typically associated with human intelligence.
***Machine learning (ML)*:** a subfield of artificial intelligence, broadly refers to the ability of a computational platform to learn from data and make predictions or recommendations based on this data without being explicitly programmed. In general, there are two major categories of machine learning, supervised and unsupervised. *Supervised learning* is conducted with the concept of “truth” where the model tries to approximate the relationship between inputs and labeled outputs. For example, given images of cats and dogs, where each image has a correct answer, can you train a model that accurately identifies of cats versus dogs? *Unsupervised learning* is performed without data labels and the goal is for the computer to infer inherent structure or patterns in the data. For example, given a set of heart rate, accelerometer, and location data from a wearable fitness monitor, can the computer identify periods of rest versus exercise based on differences in the raw data?
***Neural networks (NN):*** a form of machine learning with a basic architecture consisting of nodes and connections existing in multiple layers, loosely analogous to neurons and synapses in the biological brain. This broad category is inclusive of many kinds of modern machine learning models which are used in tasks such as computer vision, voice recognition, bioinformatics, and among others.
***Deep learning:*** A broad family of neural network architectures that have multiple layers (aka deep).

## Key Questions

For the interested clinician, these following self-assessment questions may help in determining whether a machine learning tool makes sense for your specific scenario.

### What Is My Unmet Need?

For machine learning to make a positive impact on patient care, finding the right use case is the place to start. As a practicing clinician, this should draw from your understanding of the clinical workflow and impact on patient care. A proposed paradigm is starting with a larger problem, mapping out the workflow, and identifying areas in need of improvement.

### Is Machine Learning Useful for This Need?

Critically consider if machine learning is the best tool to improve that specific area. Consider other solutions involving personnel, workflow, or policy changes. If an information technology solution is the best answer, consider its impact on the workflow in the best case scenario. This depends on what is important for each clinical scenario: accuracy, timeliness, or reliability. If even the best case scenario leads to minimal improvement and significant changes in the workflow (with attendant costs), machine learning may not be the best solution. Consider other solutions involving personnel, rules-based systems, or process redesign.

### Are You Asking the Right Question to Put the ML Tool in the Highest Value Use Within the Clinical Workflow?

In order to do this effectively, first find the right use case (e.g., right information to the right person at the right time). Next, figure out where the model fits into the clinical pathway, which includes process mapping to understand types of input data needed and output desired. Finally, consider the workflow and needs of the end-user, including timeliness.

### Should Computer Simulation Be Considered in the Development Process for the ML Tool?

Depending on the deployment setting, the ML tool may benefit from data augmentation to improve generalizability, particularly if the tool is to be applied across different radiological, electronic health record, or genomic platforms. This can be achieved with generation of synthetic data or techniques of data transformation. These are methods where data is artificially manufactured rather than the result of real-world measurement. This approach can sometimes be used judiciously to augment real-world data in scenarios where real-world data is sparse or difficult to obtain. Data can be either created de novo based on a set of criteria or by digitally manipulating real-world measured data. This approach should be used cautiously due to multiple tricky considerations including bias and generalizability.

## Case Studies

### Medical Imaging Perspective

Radiographic medical imaging, whether CT, MRI, Ultrasound, or other modality, is an ever-growing source of big data in healthcare. Medical imaging began as a field in which advanced technology was used to generate visual data that could be analyzed and assessed qualitatively by a clinician. As the field evolved, quantitative imaging metrics were developed to assist with image interpretation and management decisions (Giger et al., 2008). The advent of computer-aided diagnosis and detection in the 1980's and 1990's brought early machine learning techniques to the medical imaging field with important applications in breast cancer mammography and ultrasound (Jiang et al., 1999; Freer and Ulissey, 2001). Over the past decade, the emergence of deep learning neural networks has generated a tremendous amount of attention in the field of medical image analysis for its transformative potential (Ker et al., 2018). Deep learning utilizes raw pixel or voxel input from images and feeds them through progressively more complex layers of a neural network to generate an output prediction. Through an iterative training process, millions of mathematical parameters of a neural network are optimized such that input images fed into the network generate predictions that best fit the true output. Rather than rely on user input to pre-engineer and determine appropriate features for the machine learning model, deep learning utilizes raw imaging data to “learn” the features that optimize predictive performance. Unlike prior forms of computer-aided analysis, deep learning has the potential to form end-to-end prediction models encompassing multiple parallel or sequential imaging tasks, including object segmentation, detection, and identification. Deep learning has the potential to affect medical imaging in healthcare by (1) improving diagnostic efficiency and achieving cost savings by freeing up limited human resources, (2) augmenting human performance at diagnostic prediction in challenging scenarios, and (3) discerning previously impossible-to-discern patterns and predictions from imaging data.

#### Case Study: Lung Cancer Screening

Lung cancer is the leading cause of cancer death in the United States. Lung cancer screening with low-dose CT has been shown to reduce mortality and is currently recommended routinely for high-risk individuals (National Lung Screening Trial Research et al., 2011; de Koning et al., 2020). Despite imaging guidelines for lung cancer detection, there remains significant concern surrounding inter-rater variability, and false-positive and negative rates (Field et al., 2016). Additionally, uptake of CT screening, even among high risk populations has remained extremely low, in part, owing to lack of high-volume radiology center resources (Jemal and Fedewa, 2017). Given these challenges, there is a unique opportunity to explore machine learning to improve accuracy of detection and access to screening. In embarking on an investigation of machine learning for lung cancer screening, the following should be considered:

What is the goal and what is the metric of success? The ultimate goal and metric for success may not be the same thing, particularly in initial phases of algorithm development. The ultimate goal should reflect clinically meaningful endpoints: improving patient survival, quality of life, or healthcare costs. The metric for success often begins more narrowly. In the case of lung cancer screening, percent accuracy, sensitivity, specificity, and the area under the ROC curve in identifying a lung nodule as benign or malignant may be appropriate. Ultimately, as study progresses, metrics should move beyond accuracy. Direct measurement of clinical meaningful endpoints, such as survival, morbidity, and quality of life should be incorporated into clinical trials of the application.

What type of machine learning is optimal for the task? The type of machine learning utilized will be driven by the medical imaging task, however, in general, convolutional neural network-based deep learning architectures are the current gold standard for image analysis. In simplistic terms, a convolutional neural network takes images as input data, and applies various filters which manipulate the image to extract features. This is analogous to image filters you can use in photo manipulation software or various social media programs. Some filters may enhance borders or edges, others may detect specific colors or brightness levels. This strategy is used in a neural network where the final output is based on extracting meaningful features from the images and making decisions based on those features. Older methods utilizing pre-engineered radiomic features may be suitable for certain classification problems where the image region of interest is well-defined, but deep learning has the ability to both localize an object (in this case lung nodule) *and* classify it (malignant vs. benign*)*. Deep learning is particularly well-suited for this “end-to-end” task completion. Several studies have shown extremely high accuracy of lung nodule and malignancy prediction using a deep learning based approach to CT diagnosis (Field et al., 2016; Jemal and Fedewa, 2017; Kang et al., 2017; Causey et al., 2018; Ardila et al., 2019).

What type of data is needed? Data collection, curation, and annotation are perhaps the most critical aspects of training a successful machine learning algorithm. As the approach shifts from simpler machine learning models to more complex models such as deep learning neural networks, the quantity and quality of data becomes increasingly important. For lung cancer screening, this means access to thousands of CT scans that have been pre-labeled by human experts. Each nodule should have been identified and should have associated with it a “ground truth” label. For an image localization task, this label itself would be a segmented region of interest encompassing the nodule. For malignancy classification, this label could be binary (malignant or benign) or ordinal (suspicion of malignancy on a scale of 1 through 5), depending on how the labeling was performed. Because many imaging-based ML algorithms are prone to overfitting training data, all models must be validated on external datasets, ideally representative of the target scenario for which the algorithm is being developed. Particular considerations for medical images are type of CT scanner, use of contrast agent, image resolution, and artifact. These parameters must be explored and addressed in preprocessing steps and/or validation datasets prior to implementation of an imaging-based ML application.

What is the role of simulated, or synthetic, data? Successful ML development in medicine requires large, high-quality, annotated, and accessible datasets, which are often lacking (Emanuel and Wachter, 2019). A key strategy to mitigate data limitations is the use of data augmentation techniques to create simulated, or synthetic, data to bolster the training process. By applying image transformations, from simple rotations, flips, or deformations to more advanced ML-driven augmentation, model generalization can be improved dramatically even when training on relatively small datasets (Goel et al., 2020). This is accomplished by introducing transforms that mimic confounding variations expected of data samples encountered in real-world testing, but that are not themselves features that predict a particular data class.

Where does the model fit into the clinical pathway? The ultimate utility of an ML-based healthcare application like lung cancer screening will not be decided by AUC or accuracy, but by clinically meaningful endpoints, such as decreased mortality, treatment-related morbidity, and healthcare resource burden. To maximize the potential utility of the algorithm, it must be determined how the model can best fit into the clinical pathway by considering timing, physical space, costs, user interface, and responsibility. In the context of lung cancer screening, for example, an algorithm could be executed automatically at the time of scan or by the radiologist during review. The former could improve resource allocation by flagging abnormal scans for expedited review, but the latter would allow for human oversight of the algorithm with less risk of bias. On the other hand, incorporation at the time of radiologist review would necessitate a streamlined user interface that does not compromise efficiency. Simple workflow decisions such as this can also have profound implications for responsibility, trust, and decision-making and raise medico-legal issues. If an algorithm triages patients incorrectly to the reviewing radiologist, who is liable for this error? These subtle implementation characteristics represent significant barriers to entry to real-world clinical use, but are often overlooked in early stages of algorithm development. These factors should be considered (and reconsidered) at each stage of algorithm development, even at model conception.

### Ambulatory Provider Perspective

A major advantage of machine learning algorithms is the ability to process large amounts of data in a relatively short amount of time. For an ambulatory provider, this advantage can translate into individualized decision-making by using a model that incorporates relevant variables beyond traditional population-based risk factors. For example, primary care providers often use a clinical decision support tool to recommend initiation of a statin for appropriate patients during routine office visits. Traditional models such as the Atherosclerotic Cardiovascular Disease (ASCVD) risk score uses conventional statistical methods from a population that may not be a good representation for all patients, particularly since risk of disease and treatment guidelines vary among patients of different ethnicities (McCredie et al., 1990; Norwood et al., 2009; Lloyd-Jones et al., 2017; Das et al., 2018; Volgman et al., 2018; Damask et al., 2020).

#### Case Study: Polygenic Risk Scoring

To better understand differences between individuals of different ethnicities, polygenic risk scoring estimates the predisposition of disease using the presence or absence of known disease-associated genes (Damask et al., 2020). This holds the promise of generating more accurate predictions by using genotypic data in conjunction with other clinical and environmental variables. As a clinician interested in implementing such a model into live practice, what are the important specifics to consider?

Machine learning models can process a large number of variables that are also very different from one another. In order to handle the variety of data, data management is critical during the early stages of planning. Effective data management considers (1) data type, (2) data reliability, and (3) the sample size.

In regards to data types, the inputs used to generate a model can come in various forms. One of the major advantages of newer machine learning models over traditional statistical models is increased flexibility to take different types of inputs. This can range from simple mixing of categorical vs. continuous variables to handling high dimensional complex inputs such as raw imaging, video, audio, or even genome sequencing data. Another advantage of handling multiple data types is that one can imagine a machine learning pipeline that utilizing several layers of processing while appearing seamless to the end user. If a data type is not readily available in modern EHRs but is of critical importance, it should be considered for integration as part of future policy/health IT infrastructure development. For example, in order to fully utilize genomic risk prediction, sequencing data must be available. At present, most genomic sequencing is often done for a specific panel of genes and the results are often saved as a report in the EHR. The actual genomic data is not saved as most mainstream EHRs lack the capability to store this type of data.

When considering data reliability, the electronic health record (EHR) is a rich source of potential data but most clinicians recognize that there is a wide range to the reliability and accuracy of EHR data. Some data types are structured (for example a hemoglobin A1C laboratory value), meaning both the value and the context are discretely defined. Structured data are more easily accepted by machine learning algorithms with less preprocessing needed. These types of data fall on the more reliable end of the spectrum. Diagnosis and billing codes are also structured, in that the value and context are clearly defined, but most clinicians understand that they are limited terms of accurate representation of the patient. Fully unstructured data include data types such as notes. Notes are often considered the most representative of clinical truth in the EHR, but often can still contain errors. As unstructured data, notes are difficult for machine learning models to accept as input without preprocessing.

A challenge for generalizability in polygenic risk scores is the heterogeneity of available data across electronic health record systems that vary across institutions and health systems, and scarcity of fully annotated genomic datasets. Two promising approaches have shown the ability to generate synthetic data by characterizing different data distributions in electronic health record data and genomic datasets using generative adversarial neural networks and ordinary differential equation-based models (Fratello et al., 2015; Baowaly et al., 2019).

A critical question for data scientists is identifying what types of data is important to include into a model. Clinicians can inform data scientists about important concepts to include and point them to the best sources of data to represent those concepts. This often draws on the clinician's medical knowledge and may mirror their own human analytic process when making clinical decisions. For example, clinicians understand that diabetes is an important risk factor to include in models for cardiovascular disease. Therefore, an important concept to identify is the presence of “diabetes.” However, data that could represent diabetes include laboratory values, clinical documentation, billing codes, and among others. The ultimate decision on which to use (including combinations) is best made in conjunction with a clinician who understands the medical considerations, the workflow considerations, and the data considerations as discussed above. Once there is a thoughtful strategy on what are the best sources of data for specific concepts, advanced ML techniques can be employed to obtain more difficult to extract data if necessary.

For example, a common challenge in utilizing the EHR is how to best utilize clinical notes, where information largely exists as free-text. One approach to make use of unstructured free-text data is natural language processing. Natural language processing models can capture specific meaning and interpret intent based on not just terms but context. A combination of ML models optimized for specific tasks can be integrated into a larger model either directly or through a series of preprocessing steps where the output is used in a subsequent ML model. Specific ML models may be optimized for natural language processing of notes, or detect polygenic risk of cardiovascular disease from genomic data. In the prior example, NLP may be used to identify the concept of “poor adherence to insulin” from clinical notes whereas a genomic risk factor model may find mutations that confer risk of developing type II diabetes. These data points can subsequently integrate into a model that accounts for environmental exposures such as smoking and other clinical risk factors like obesity.

The last major consideration is the number of cases or patients with the relevant data available. While most of the current excitement is over deep learning or neural networks, these types of machine learning techniques require large numbers of examples to train. Other forms of machine learning can perform well with smaller training samples, and some approaches handle missing data better than others, which is a frequent occurrence when working with clinical variables. Lastly, understanding the population characteristics can be helpful when selecting good machine learning model candidates. Like all prediction modeling, incidence and prevalence are important considerations when attempting classification tasks. For example, rare events can be more difficult for machine learning to predict, and data scientists often address issues related to class balance when building the model. If a machine learning model tried to predict whether you would win the lottery, and just predicted that nobody would win the lottery ever, it would be right the majority of the time and be very “high performing” from an accuracy perspective.

### Proceduralist Perspective

Real-time deep learning-based computer vision can also enhance the performance of the proceduralist by providing visual enhancement of anatomy and pathology. These can be overlaid directly onto images collected during the procedure, whether from laparoscopic surgery or diagnostic endoscopy. The algorithms could provide optical biopsies, map out anatomical boundaries and tissue planes, and identify abnormal areas relevant to the particular operative procedure.

#### Case Study: Colonoscopy With Computer-Aided Detection

For a gastroenterologist, finding precancerous lesions is the top priority to prevent colorectal cancer. In order to measure the success in preventing colorectal cancer that develops before the recommended next colonoscopy, gastroenterologists have traditionally used adenoma detection rate (ADR) as a proxy indicator for high quality colonoscopy as a 1% increase in ADR in correlated with a 3% decreased risk of interval colorectal cancer. The endoscopist can track their adenoma detection rate, and if it is lower than expected could undergo additional training to improve their ability to detect pre-cancerous lesions. However, adenoma detection rate has wide variation across endoscopists, and a tool that would standardize the performance of endoscopists would help decrease the incidence of preventable colorectal cancer. Recent advances in deep learning through convoluted neural networks have led to high-performing algorithms that hold promise in enhancing endoscopist performance by identifying polyps in real-time colonoscopy videos and detecting adenomas, which can increase the adenoma detection rates for all endoscopists (Misawa et al., 2018; Urban et al., 2018; Wang et al., 2018, 2019, 2020; Gong et al., 2020).

As a clinician interested in developing or implementing deep learning tools to improve the adenoma detection rate, how should you think about the approach?

First, identify the inputs (e.g., images or video) to train the model, which in this case would be deep convolutional neural networks described earlier in section Medical Imaging Perspective. If the model is meant to detect polyps, the ideal input would be colonoscopy videos with labeled images of “polyp present” and where in the frame the polyp is located. This is the rate-limiting step, since labeling is human capital-intensive, deep learning requires numerous examples, and the algorithms learn explicitly from the label of “polyp present” or “polyp absent.”

In this particular task, data transformation has been considered due to the relative absence of large annotated image databases of polyps. These approaches have included changing the image dimensions, changing pixel values, and adding in external conditions with the goal to maintain or achieve better generalizability for ML tools to detect polyps (Sánchez-Peralta et al., 2020).

As with all supervised machine learning, labels must be present in the data to train the algorithm, which can sometimes be costly as content experts are needed to create the labels. Furthermore, one key challenge is to make sure the algorithm can perform well in other datasets, referred to as “robustness,” such as in real time for a new procedure. In this case, the specific way the data is captured may affect the algorithm performance. For example, if the algorithm is to be used in a practice with high definition endoscopes that have specific image processing settings (e.g., narrow band imaging), the input data should ideally be captured from that specific brand of endoscope and also include images with the specific setting. A clinician is critical in informing the data scientist the parameters of the data used during the procedure so that adequate data of sufficient quality is collected to train the algorithm.

As the model is developed, the issue of timeliness and workflow is highlighted as a key area for clinician involvement (Shung and Byrne, 2020). This is particularly relevant for endoscopic units in ambulatory surgical centers, where the trend toward lower reimbursement for endoscopy have led to the development of performance metrics to enhance efficiency (Gellad et al., 2013). Proceduralists provide crucial information about the existing clinical process to guide how software should be designed. The user needs of the endoscopist must be considered, particularly the tolerance for false positives and the impact of the software on efficiency (i.e., duration of the procedure). Since a colonoscopy procedure involves diagnosis, assessment, and treatment (find the polyps, assess if they are problematic, and remove them), real-time processing is a prerequisite to any software solution. For high volume ambulatory surgery centers, algorithms must have minimal impact the amount of time to perform procedures. Clinicians' preferences and insight into the workflow of how the deep learning software enhances the user experience and performance are key in optimization, in this case providing real-time recommendations that do not unnecessarily prolong the overall procedure.

Finally, as these algorithms are implemented, clinicians have an important role in providing feedback. User assessments and improvements in the interface for each iteration of the software implementation. If there are clear discrepancies in what the software detects and provider assessment, quality control is crucial to maintain provider confidence in the software recommendations.

#### Limitations and Additional Considerations

While these scenarios delve into specific ways in which clinicians can inform the development and validation of ML tools in clinical care, the potential applications of AI in healthcare go from individualized recommendations with personalized medicine to informing policy in public health. A discussion of all the potential applications is beyond the scope of this article, but a comprehensive compilation of AI and ML-based medical devices approved by regulatory bodies in the United States and Europe provide a glimpse into the personalization of care (Muehlematter et al., 2021), while another article delves into the ways in which AI and ML can be used across populations to tailor policies to promote health, protect health, and improve the efficiency of services for communities within the greater population (Panch et al., 2019).

Limitations for integrating ML tools into clinical care can broadly fall under data maintenance and real-world deployment.

Bias, heterogeneity, and gaps in data can lead to poor performance or contribute toward perpetuating disparities or harmful discriminatory practices. Indeed, a prominent recent example was the Amazon AI recruitment tool that was deactivated after it showed bias against hiring women (Dastin, 2018). A new concept of algorithmic stewardship addresses the limitations of constantly changing sources and storage of healthcare data by monitoring, correcting, and updating the dataflow to accurately reflect different ways of data capture as well as practice patterns or epidemiological shifts (Eaneff et al., 2020). Data equity and representation is a key limitation that should be actively addressed with the development of any ML tool to ensure that inherent health inequities, such as race correction, will not be perpetuated (Vyas et al., 2020).

Generalizability and interpretability are two key limitations that can hamper real-world deployment of ML tools. For clinicians, the focus is on the individual patient, which requires that the algorithm performs well and does not generate an erroneous result. For deep learning tools in particular, the key limitation of overfitting due to complexity of the network architecture and large number of parameters must be addressed with rigorous validation on multiple datasets representative of real world data. This is analogous to training a robot to play tennis only on a clay court, and then deploying the robot to play on the grass courts of Wimbledon. Since clinicians are experts with advanced training, the need to trust and verify the ML tool output is key to ensure that the ML tools are used in clinical practice. For this, a measure of interpretability is important so that ML tools can complement the professional authority of clinical providers (Kelly et al., 2019).

The use of AI with its dependence on data also introduces additional risks into the healthcare environment with regards to ethical, regulatory, and legal issues. Privacy compliance, the role of the algorithm in shared patient-provider decision making, data access, system failures, computer viruses/malware, and intentional adversarial attacks geared toward machine learning models require additional strategies to mitigate risk for patients when considering the use of ML in medicine (Finlayson et al., 2019). Ethical research methodology, including fairness and equity for both representation in the data used for the algorithms and in sharing the benefits realized by the algorithm, must be practiced when using patient data. Clinician researchers adept in these consideration can help guide data scientists in this regard. We recommend consultation with institutional review boards (IRB) for all projects related to patient data to ensure appropriateness and proper protection of patients. Prior to commercialization and deployment of informatics based tools in patient care, approval from regulatory bodies may be necessary. The regulatory guidelines continue to evolve in the United States with the FDA, the European Union with General Data Protection and Regulation framework, and internationally through the International Medical Device Regulators Forum. For the FDA, ML algorithms have been assessed in a similar fashion to medical devices, although there is now a growing recognition that software-based products are a unique category within that track.

## Conclusions

Machine learning integrated medicine is the future of patient care. Analytic tools to take full advantage of an increasingly information-dense practice environment, but clinicians are critical partners in developing successful ML models that can be integrated into real-world patient care. While data scientists are experts in the technical aspects of machine learning, clinicians are needed to identify the appropriate settings for ML solutions, the best data to use to help shape model development, the best integration point into a real world workflow environment, and the final usability of the tool.

## Author Contributions

LS came up with concepts and critically revised the manuscript. BK wrote substantive parts of the manuscript and critically revised the manuscript. RT critically revised the manuscript. DS wrote the manuscript, contributed concepts, and critically revised the manuscript. All authors contributed to the article and approved the submitted version.

## Conflict of Interest

The authors declare that the research was conducted in the absence of any commercial or financial relationships that could be construed as a potential conflict of interest.
